# Tobacco and mental health- Deciphering the vulnerability to nicotine in patients with schizophrenia as a function of their dopamine neurotransmission genes: a clinical and preclinical study

**DOI:** 10.1192/j.eurpsy.2023.1946

**Published:** 2023-07-19

**Authors:** J. Mallet, V. Gorgievski, N. Ramoz, E. Tzavara, C. Dubertret

**Affiliations:** 1 Psychiatry, APHP, Paris; 2 Psychiatry, APHP, Paris, France

## Abstract

**Introduction:**

Schizophrenia (SZ) is a severe and frequent mental disorder that has multifactorial origins (genetic but also environmental vulnerability, gene environment interactions). Our team has shown that in France smoking is common among these patients and often begins before the onset of symptoms. This calls into question the hypothesis of self-medication of psychotic symptoms and cognitive disorders by tobacco consumption, and raises the question of specific interactions between nicotine consumption and the genes of the dopaminergic and cholinergic nicotinic systems, in particular with regard to adolescence, period of neurodevelopmental vulnerability.

**Objectives:**

to study (i) the interactions between the DA system and exogenous nicotine in a pre-clinical mouse model (transgenic for DAT) (ii) the impact of smoking on the psychotic and clinical phenotype in a national cohort of SZ patients (iii) interactions between tobacco consumption and several genetic polymorphisms of the dopaminergic and nicotinic system in SZ population. Hypothesis: Disruption of the balance between DA and nicotine systems by nicotine consumption in adolescence may be a key neurobiological mechanism for the emergence of SZ disorders.

**Methods:**

The characterization of the model is behavioral (anxiety, memory, social interactions, locomotion, motor coordination) but also biochemical. For the clinical approach, we exploit the clinical / cognitive / genetic data of a national cohort (Fondamental foundation) and another smaller and local cohort.

**Results:**

We demonstrate, for the clinical study, that some clinical and cognitive characteristics are associated with tobacco use in schizophrenia patients, with more cognitive distubrances in smokers, against the self medication hypothesis. Genes envionment interactions also demonstrate associations with genes involved in dopaminergic system. Regarding the preclinical study, we show a gene environment interaction, as heterozygote mice for the DAT gene exposed to Nicotine durig a critical neurodevelopmental window ( adolescence) show a loss of familiarity in the social memory test and a loss of cognitive flexibility at the set shifting test, similar to what is found in schizophrenia.

**Conclusions:**

TBetter characterization of patients with schizophrenia is necessary to better understand the physiopathology of this disease and explore new personalized preventive and therapeutical targets. We show that tobacco is associated with cognitive disturbances in schizophrenia patients, against selfmedication hypothesis; and in our animal model, that nicotine exposition during the adolescence combined to a moderate cortical and limbic hyperdopaminergia, is associated with persistent cognitive and social deficits, in favor of a gene environment interaction.

**Disclosure of Interest:**

None Declared

